# Iranian researchers’ perspective about concept and effect of open science on research publication

**DOI:** 10.1186/s12913-023-09420-9

**Published:** 2023-05-04

**Authors:** Maryam Zarghani, Leila Nemati-Anaraki, Shahram Sedghi, Abdolreza Noroozi Chakoli, Anisa Rowhani-Farid

**Affiliations:** 1 Department of Medical Library and Information Sciences, School of Health Management and Medical information science, Iran University of Medical Sciences, Rashid Yasmin Street, Upper than Mirdamad St, Tehran, Iran; 2 Health Management and Economics Research Center, Iran University of Medical Sciences, Tehran, Iran; 3 Health management and Economics Research Center, Health management research institute, Iran University of Medical Sciences, Tehran, Iran; 4 Department of Information Science & Knowledge Studies, Shahed University, Tehran, Iran; 5 Department of Pharmaceutical Health Services Research, University of Maryland School of Pharmacy, Baltimore, MD USA

**Keywords:** Open science, Openness, Openness in Research, Open Research, Open Science Practices

## Abstract

**Background:**

Sharing research outputs with open science methods for different stakeholders causes better access to different studies to solve problems in diverse fields, which leads to equal access conditions to research resources, as well as greater scientific productivity. Therefore, the aim of this study was to perceive the concept of openness in research among Iranian health researchers.

**Methods:**

From the beginning of August to the middle of November 2021, twenty semi-structured interviews were held with Iranian health researchers from different fields using purposeful, snowball, and convenience sampling. The interviews continued until data saturation. Data analysis was performed with thematic analysis using MAXQDA 20. Finally, seven main issues related to open science were identified.

**Results:**

Through analysis of the interviews, 235 primary codes and 173 main codes were extracted in 22 subclasses. After careful evaluation and integration of subclasses and classes, they were finally classified into nine categories and three main themes. Analysis showed that openness in research was related to three main themes: researchers’ understanding of open science, the impact of open science on publication and sharing of research, concerns and reluctance to open research.

**Conclusion:**

The conditions of access to research output should be specified given the diversity of studies conducted in the field of health; issues like privacy as an important topic of access to data and information in the health system should also be specified. Our analysis indicated that the conditions of publication and sharing of research processes should be stated according to different scopes of health fields. The concept of open science was related to access to findings and other research items regardless of cost, political, social, or racial barriers, which could create collective wisdom in the development of knowledge. The process of publication and sharing of research related to open access applies to all types of outputs, conditions of access, increasing trust in research, creation of diverse publication paths, and broader participation of citizens in research. Open science practices should be promoted to increase the circulation and exploitation rates of knowledge while adjusting and respecting the limits of privacy, intellectual property and national security rights of countries.

## Introduction

Governments and funding agencies in most countries have supported the idea of openness as a vital component in scientific research through open access policies. Open science broadly refers to the sharing of resources, ideas, and other outputs from research with an emphasis on making these resources publicly and freely available for maximum usage, making science more open, accessible, global, transparent, integrated, and collaborative [1]. Open science practices are applicable by promoting the reuse of data, increasing accuracy and replicability, reducing redundant researches, facilitating the sharing of processes and results, improving communication with a greater variety of actors to produce the innovative approaches and solutions in medium and long term [2], which result in the validation of science as well as higher efficiency in response to challenges of the society [2, 3]. In fact, a new approach has been defined for the scientific process based on team work, involving new pathways for collaboration and publication of knowledge through online digital technologies [4, 5]. This approach removes barriers of sharing the scientific research outputs [6], which can take many forms in the research process, including the insertion of data into online databases or journal repositories, developing international standards for formatting, organizing, and modifying data, publishing in open access journals, creating software, models, or materials that can be used in projects, laboratories, and different fields [7].

The successful performance of open science can be influenced by factors contributing to the production of new knowledge, including attracting young scientists in research-related jobs, developing appropriate ways to evaluate the quality of research, presenting grants, and guaranteeing the integrated culture of research [1, 8]. In other words, open science addresses new incentives and methods to be more compatible with democratic rights and values in access to knowledge, as well as the development of open tools for collaboration for bridging the gap between science and society [9]. This could contribute to the progress of humankind though the promotion of sharing scientific ideas, concepts, data, code, methods, and results not only for researchers but also for consumers, industry, and society in general [10].

Considering the importance of access to data and other research outputs, a number of organizations, funders, journals, and scientific communities in different countries have taken steps to formulate open-science policies and guidelines that go beyond open access publications. RCUK’s policy focusing on “unrestricted online access to peer-reviewed and published research articles without any access fees“ [11], Access and Reuse of Research Data Statement provided by Horizon 2020, the principle of “as far as possible it should be open, closed if necessary” have been suggested in this regard; data management programs should be mandatory even if the research data is not openly available [12]. Union of European Research Universities published a roadmap for open data management [13]. San Francisco Declaration called for a set of fundamental changes in the evaluation and publication of knowledge based on open science techniques [14]; assessment methods, incentives, and services needed to support open-science and research have also been presented in Declaration of Open Science and Research of Finland in 2020 [15].

Moreover, several aspects of open science and their effects have been investigated in numerous studies, including positive effects of open science techniques on increasing trust in science [16], facilitating access to research products and outputs, promoting the participation of different individuals in research activities [17], filling the gap between science and politics [18], and making knowledge more democratic [19]. Also, methods such as pre-registration of study hypotheses, linking to statistical codes, and explicit sharing of data may result in reliable and valid conclusions [20]. For open science methods to be more productive, a change of direction is required from the standard practice of publishing research results in scientific journals to sharing all available data and knowledge in all stages of research process. This requires moving from “publication as soon as possible” to “sharing knowledge as soon as possible” [9].

For several reasons, transition from “publication as soon as possible” to “sharing knowledge as soon as possible” has gained reputation among different stakeholders and increased equality in access to different research resources, as well as participation, innovation, and productivity of science among different groups. Open science is a human rights issue, which is exquisitely included in Article 27 of Universal Declaration of Human Rights in 1948 [9]. Considering the significance of openness in access to research products and scientific policies of Iran based on maximum access to valid knowledge in health-related decisions, it is important to evaluate the viewpoints of researchers in relation to openness in science and what they consider to be the best method. This research deals with the concept and effect of open science on health research, especially in relation to the processes of publication and sharing of research outputs from the perspective of researchers and its impact on their working conditions. Considering the importance of this topic in scientific works, the aim of this study was to evaluate the concept and effect of open science regarding the process of publishing health studies, which has not been studied in Iran so far. The insights presented in this study can highlight the effect of open science on research to create a clearer perspective for the stakeholders in health-related fields.

## Materials & methods

### Participants

This is a qualitative study, the population of which consisted of all health system researchers who experienced research management in Iranian Ministry of Health (MOHME) and its affiliated universities. Snowball, purposive and convenience sampling were used to determine the sample size. The inclusion criteria were as follows: (1) at least 3 years of research experience (2) at least one year of research experience in a dimension of open-science such as open access, open data, etc. (3) managerial or executive responsibility as vice president of research in Ministry of Health; and (4) willingness to participate in the study. According to these criteria, the interviews continued until data saturation, which finally led to 20 interviews. The saturation process means continuation of interviews until the researcher does not obtain new data related to the research topic and receives repetitive data. The research environment was specified in the workplace of academic staff members and managers for in-person interviews. The interviews were held using phone call or the desired social media platform (such as Skype, WhatsApp,). The author contributions to this study were as follows. The interviews were collected by M.Z; implementation, analysis, and initial draft were done by M.Z and L.NA. A.RF, A.NC; and S.S contributed to preparation of the final version and implementation of the study.

### Data collection

A semi-structured interview guide was used to collect the data, which was prepared according to the purpose and background of the study. The questions were related to the concept of open science, open access and researchers’ experiences of the effect of open science on publication and sharing processes of research outputs (Additional file1.IIR). Firstly, during the interviews held with five participants, the number of questions, timing of interview, and the interview guide were finalized. The length of each interview was 60 to 90 min. The interview period was from the beginning of August to the middle of November 2021. After identifying the study population, our research team attempted to involve researchers from different fields of health sciences with research work experience in the interviews so that the diversity of participants increased the reliability and verifiability of the data. After the initial agreement with research samples to determine the date, time, and method of interview (in-person or online), the schedule for interview and interview guide were sent to them via e-mail. Interviews were held in three ways: in-person, telephone, and online using media social platforms such as Skype, WhatsApp, or other similar software that enabled easy conversation. Finally, 10 in-person interviews, 5 telephone interviews, and 5 online interviews were held. The interview process was explained to participants to obtain their informed consent by ensuring the confidentiality of information, and the informed consent form was completed at the beginning of each interview. The study procedure was approved by Medical Ethics Committee of Iran University of Medical Sciences [date: Jul 2020, ID: IR.IUMS.REC.1399.462], and the study only included those who presented their informed consent. For this purpose, informed consent form (Additional file2.ICF) was completed by all participants after explaining the study goals. The participants agreed with recording their voice and taking notes, and also they were assured that they could withdraw from the study at any time. Besides, (P1.P2) coding was used to specify and arrange the interviews in order to ensure the confidentiality of participants’ personal information.

### Data analysis

Data analysis was done using thematic analysis. After holding each interview, the interview was first heard several times by the researcher (i.e., the researcher responsible for holding the interview), and then its text was transcribed using Word 2013. The transcribed text was read several times, and the primary semantic units were identified. The transcribed files were transferred to MAXQDA 20, and the primary codes were determined and analyzed. Thematic analysis method was used to classify codes, extract and categorize the main categories and subcategories. Interviews were analyzed during data collection so that the researcher could master the process of interviews based on research objectives. To assess the validity and reliability of data, the participants reviewed the data and the researcher was continuously associated with the data. In the review performed by participants to confirm the validity, a part of the transcribed text and the initial codes were sent to some of them to compare and confirm the consistency of ideas emerging from the data with their own materials. To control the validity of data, the degree of agreement among the three coders was measured; they coded the first five interviews in parallel, and then the agreement among the codes was discussed. At the end of initial analyses, the person responsible for transcribing and analyzing the interviews reviewed the interview texts again and merged similar codes and subcategories. In the next step, the relationship between the subcategories was specified, and they were classified into larger categories. Finally, the main subjects of the study were extracted.

**Table 1:** Demographic information of participants

Participants’ No.	Gender	Specialization	Academic degree and job position	Participation tool in the interview
P1	Male	Health and treatment services’ management	Associate professor and vice chancellor for research and technology and researcher	Phone call
P2	Male	Librarianship and medical notification	University lecturer and researcher	Virtual - Skype
P3	Male	Health and treatment services’ management	Associate professor, researcher, vice chancellor for education	Phone call
P4	Female	Epidemiology	Research professor - researcher - director of research center	Phone call
P5	Male	Immunology	Associate professor-researcher	Virtual - Skype
P6	Male	Medical ethics	Associate professor - researcher - secretary of the ethics committee	Phone call
P7	Female	Psychiatry	Professor-researcher	Phone call
P8	Female	Pregnancy health	Professor-researcher	Virtual - WhatsApp
P9	Male	Professional health	Research professor - researcher	In-person
P10	Male	Physiology and neuroscience	Professor-researcher	In-person
P11	Male	Radiology	Associate professor-researcher	In-person
P12	Male	Medical bioinformatics	Assistant professor-researcher	In-person
P13	Male	Epidemiology	Assistant professor-researcher	In-person
P14	Male	Epidemiology	Assistant professor-researcher	In-person
P15	Male	Professional health	Assistant professor-researcher	In-person
P16	Male	Health information management	Assistant professor-researcher	In-person
P17	Male	Biochemistry	Assistant professor-researcher	In-person
P18	Male	Health information technology	Assistant professor-researcher	In-person
P19	Female	Librarianship and medical notification	Assistant professor-researcher	Virtual - Skype
P20	Male	Biotechnology	Assistant professor-researcher	Virtual - Skyroom

## Results

A majority of participants were males (16 out of 20). All of them were active researchers in the field of health; in addition to carrying out research studies, they worked as active professors and lecturers in medical universities. In addition to being active professors and researchers, four participants were decision-makers in the field of health system research. The demographic information of interviewees is given in Table 1. A total of 235 primary codes were reviewed and merged, resulting in 173 main codes. A total of 173 main codes were extracted in 22 subclasses, 9 categories and three main themes (Table 2). The main themes were as follows: researchers’ understanding of open science, the impact of open science on publication and sharing of research, concerns and reluctance to open research.

**Table 2:** Subjects related with open science

Health science researchers’ understanding of open science	The impact of open science on publication and sharing of researches	Concerns and reluctance to open-research
Access to research findings without any obstacles	Open access to a variety of research outputs	Prolonged review and applying personal opinions
Applying collective wisdom in research	Conditions of access to outputs	Loss of idea before final printing
	Increasing trust in research	
	Creating diverse publishing channels	
	Citizens’ participation in research stages	

### Health science researchers’ understanding of open science

The concept of open science or openness in research from the perspective of researchers is related to access to findings, data, details and other research items regardless of barriers like cost, political status, social class, race, etc. However, in addition to this maximum access, the principles and regulations protecting the rights of researchers and other stakeholders must be considered. In general, based on the experiences and opinions expressed by participants in connection with the concept of open science, we were able to identify two general categories in this regard.

### Access to research findings without any obstacles

Access to research outputs without potential and actual barriers should be checked first. In the health system, in particular, items related to the privacy of individuals cannot be freely shared without any rules and regulations. However, sharing and access to maximum output are crucial for health system research and decision-making. However, this access should be provided according to the type of audience by adjusting the identity terms of the data and information. All the participants emphasized maximum output access, especially for a country like Iran that faces the challenge of political sanctions. If this access to research is applied to an international system without paying attention to political issues, it can have good results. However, the principles of security, privacy, and rights of researchers must be observed and relevant costs specified. One of the participants says:

*“I think not all the resources provided to the research team require special permission nor special steps to access them; for example, access to sources, articles, the data referred to as secondary data, and raw data that can be prepared and used” (P. 3, Line 5–7).*

The interviewees emphasized that their perception of open science in research means access to sharable outputs of research without obstacles; at least this access should be provided to researchers and decision makers in the field of health without barriers. Also, researchers themselves should publish their findings and other research outputs in order to share the knowledge gained, improve the health system and prevent repetition. Easy access to the knowledge obtained from research shows that the researcher does not belong to a particular society or thought, and as a result, the output of his/her research also belongs to the entire society because the ecosystem of communities and health is interconnected like chains. If a problem occurs, it should be solved using the results of research in other parts of the world. These results should be shared with other sectors without a sense of scientific ownership to solve the problems and prevent further consequences. For example, one of participants says:

*“Knowledge belongs to God and it is infinite. Humans see an angle of this knowledge with the help of research that gives better insight, and its ownership does not belong to that person, that is, he/she should not sell it unless we make a product and present it (P1. ,Line37-39)” “I believe that open science means providing knowledge gained from research findings that allows for free access to research findings without political, social, economic, and cultural barriers, which is not just a matter of cost, and open-access to human knowledge means that a scientist belongs to the whole world, regardless of place of birth, and the knowledge gained belongs to the whole world” (P.1, Line 44–47).”*

### Applying collective wisdom in research

The application of collective wisdom in different stages of research is a special condition that open science can create in research process in the health system. Collective knowledge can be created based on sharing different stages and parts of research through open access. In this process, both specialized and non-specialized opinions can be consulted. From the participants’ point of view, collective wisdom has been successful in several sectors like startups and could have positive results if used in health system research, which is often interdisciplinary:

*“When research is open, well, I think collective wisdom will reduce research errors and increase reproducibility” (P. 20, Line 21).”*

Research is the product of teamwork; therefore, people from different fields should participate in a research project of the health system. This indicates that the research work is formed based on collective knowledge. In the field of health, and especially therapeutic cases, the research goes through different stages to reach a practical answer. Therefore, it is necessary to study various stages with the vision and knowledge of different minds to find the strengths and weaknesses and finally reach a more valuable effect. Accordingly, if various stages of research are based on practical openness, the use of collective wisdom in these stages is one of the most important contributing factors. For example, one of researchers reported that he experienced the application of collective knowledge in startup work. If this factor is extended to research, it will lead to the development of research ideas:

*“I agree that we can share research from the very beginning of the idea formation. There was a startup center. In weekends, they used to hold a meeting, and people from different specialties gathered there and developed the idea through ideation. If this trend is developed in research without getting worried about misuse of ideas, it will lead to the maturity of the idea” (P. 20, Line 6–9).*

### The impact of open science on publication and sharing of research

Researchers have considered the principle of open science in facilitating the publishing and sharing of research outputs. In the analysis of interviews, the type of items that can be published, the acceptable trends and the level of access to outputs have been stated for sharing the outputs. As a result, we presented the topics raised in five general categories.

### Open access to a variety of research outputs

The final article or report is the most well-known output that most participants emphasized to be published and shared. In addition, other items such as research data, tools, and software, details of research process, especially in laboratory studies, as well as measures taken in certain conditions and standards like documenting the processes, details, and conditions for conducting research were assumed. Regarding the publication of data and the process documents, few participants spoke thoughtfully about their publication and sharing; they believed that the conditions for the protection of these cases, the rights of researchers, and especially privacy issues, should be taken into account. Another significant matter considered by participants was that open access did not mean just access to journal articles, but to the availability of outputs other than the final findings or the article they were referring to, including data, software, details, methods, etc.

*“Research details should be fully published. I think this could prevent hasty decisions such as research into drugs produced for coronavirus disease” (P. 20, Line 15–16).*

The study protocols in review studies or clinical trials, research details, and even an operational plan for conducting research developed during investigations can be published. These items can have a great value for further research and decision-making, and for better assurance and effectiveness of the research, everything that can be shared and published should be available to the stakeholders. Another participant says,

*“The interesting thing is that when I started the research, they showed me a shelf containing flashcards and notebooks where all the details, position, and working conditions had to be recorded in detail, that is, the process needed to be precisely documented, and at the end of the work, you had to deliver a copy to the lab or research center and take a copy for yourself, so that if a question or issue arises, one can provide the correct answer by referring to this note. I remember working on a technique that I had a problem with, and a researcher at Harvard had conducted it, then I talked to him and he noted a very small point so that I came up with an answer, i.e., recording the details of the research is much more important than the research results in some suspicious cases” (P. 10, Line 19–26).*

From the participants’ viewpoint, sharing of working methods and conditions in medical sciences studies conducted in a laboratory or under special standards and conditions (especially in pharmaceutical protocols) bears important outputs, which can be useful for future studies, as well as trustworthiness and accuracy of the research. In addition, this sharing also generates financial income. The publication of research protocols, especially in the field of medicine, can be highly efficient for other researchers and universities. Given that the design of these protocols is time-consuming and must be highly accurate, research protocols are among the most important factors contributing to access and sharing conditions. One of the participants says,

*“Nanoparticles are difficult to synthesize or validate. If these protocols are published, they can generate both revenue and great benefits for other researchers to save costs. In my opinion, there should be documentation or a method to record and publish these cases of research outputs that can be helpful in the field of health. For example, a company in Mashhad generates revenue using this approach. They modify methods and protocols in the field of pharmacy and make them available to drug manufacturers” (P. 17, Line18-22).*

Access to all types of research data is considered an important output of research. The infrastructure and rules should be considered for publishing research, as well as public, and government data, other data necessary for research, decision-making, and policy-making. Although most participants emphasized research data, there was difference between data and the conditions for accessing them, especially health data for which privacy is a highly important issue. Data are also a power for any organization, country or researcher, and when these conditions are shared, they can provide the basis for knowledge exchange to make important decisions. One of participants says,

*“In my opinion, the data from the studies should be published, i.e. to release data along with the results, namely the data on which the study is based. Access for researchers should be securely defined in data banks. Data are collected in a bank or repository like cohorts or registry systems. Large amounts of data are collected in them. The teams that collect data do not meet the conditions to publish all of them. Other researchers’ access to this data must also be defined. Everyone analyzes this data in some way or other, and the value of the data increases in this way (P. 6, Line 36–40).*

### Conditions of access to outputs

Determining the level of access to outputs is an important matter that must be considered in the process of publishing research outputs. To release the outputs, access conditions must be determined based on the type of outputs and addressees, which is an important point in health studies. It is better to determine the protocol or basic rules for the conditions of access to outputs by researchers at the beginning of research, so that the level and amount of sharing can be determined according to the type of research and its outputs. This does not mean limited access, but rather the method of access to outputs should be determined according to the conditions of study, time dimension, and other effective factors.

Access to research outputs should not harm anyone, and the rights of people under study, as well as researchers and relevant organizations must be respected. Another issue with specific importance in the health system regarding access to outputs from the perspective of interviewees is that the collective interests of communities take precedence over those of the individual or organization and that the publishing process should be in accordance with the interests of communities:

*“For example, we produced a vaccine. Can we trade with this? Or no, should we offer it to everyone? Here, human and moral rights are a priority, i.e., we have to decide in the best interest of societies. In the field of health, priority should be given to the interest of societies. A scientist sells the formula of a vaccine for a dollar and says that it belongs to people’s health that cannot be traded” (P. 1, Line 51–54).*

The publication of output should be proportionate to its specific audience to be effective and not lead to problems like misinterpretation of research. That is, in defining the conditions for access to outputs, one of the factors that should be considered is the research audience. This process can be undertaken by special working groups. Each stage of research and sharing of outputs is monitored, and access conditions are specified. Analyses based on interviews revealed that each output should have its own audience and that access should be provided to the relevant group. Not all people need to have the same access conditions. Of course, this in itself should not lead to discriminatory conditions for access based on tastes or relationships. Access can be facilitated if the relevant protocol is set at the beginning of research design. That is, the tools, methods, or protocols are available to experts and researchers, the results are accessible to those familiar with the language of audience, or the data are presented to researchers and decision-makers. One of the participants says:

*“If this access is based on an addressee’s knowledge, it can be highly useful, and efficient analysis can be obtained. Different discussions can be done on various studies; these results are helpful, and as long as there is no access and use of data, it has no value. Sometimes data disappear and no analysis can be performed on them” (P. 3, Line 60–62).*

The main reason for classifying contacts for access to outputs is (1) positive impact of output and (2) protection of the rights of all stakeholders, namely those who conduct the research and those who are users. In this regard, one of the participants says,

*“I think that the publication of outputs should be audience-oriented and phased or made available to the public under appropriate conditions, but the important thing is to see how effective that study is; if the purpose of the research is to solve a problem, it should not cause problems due to the publication at the wrong time (p15, Line 39–42)”.*

### Increasing trust in research

Trust and confidence in research outputs can be increased through transparency and reproducibility. In fact, the main point is the trust in research outputs for application, decision-making based on science development, emphasizing the facts presented by the research, to which factors of transparency and replicability contribute. Providing such conditions is one of the most important tasks of researchers. In their view, these conditions will be realized by the publication and sharing of data, details, methods, and other research outputs. Even for this purpose, the output should be reported to a specific audience systematically when conducting research projects, especially comprehensive and large projects requiring a lot of time and special conditions. This will lead to better evaluation and provides conditions for conducting the research as well as public trust in research. For example, in pharmaceutical debates, the production of a vaccine is a challenging issue from the beginning to the end, and the community’s trust reflects in the use of the vaccine. Participant 1 says:

*“Let’s use the example of vaccination. The process of vaccine production should be reported step by step through various tribunes and provide maximum transparent information to the people, observing the legal issues and the scientific capacity of society. In my opinion, for research transparency, open science appendix or any other name appropriate for each study should be defined at the time of grant allocation, and the conditions for open access should be stated, so that anyone can provide the conditions for research transparency and replicability with the defined model and method” (P. 1, Line 84–89).*

The reproducibility of research makes the results valid and does not mean that other researches should do the same, but rather provides the conditions for evaluation, testing, and more precise studies, as well as terms for studies to be conducted by secondary researchers. Participants also reported that this factor could contribute to health system studies, and reproducibility provides conditions for other researchers to learn from previous researches in order to reduce previous mistakes and errors. For example, one of participant says,

*“Reproducibility is highly important in the field of health for promoting community health as well as resolving research weaknesses. The method must be clear and transparent; data and tools should be clearly expressed to be reproducible. If there is a problem, reproducibility can correct the mistakes of previous works and promote research on health (P. 2, Line 37–40).“*

### Creating several publishing channels

If we intend to conduct research in the framework of open science, the implementation process, the publishable and shareable items, and the access modes to these outputs must also be determined by researchers and research supporting organizations at the time of specifying the research protocol. By defining this process, the rights of stakeholders can be protected, and selecting the best approach to sharing research outputs can lead to greater productivity of research using appropriate publication methods. For example, one of participants reported that each research finding is a message that should be in the right publication direction and be available to its specific audience. Another participant reported that all outputs should be published. Now, it must be decided at what position it can be released depending on the type of output,

*“Each research finding is a message, the right way of spreading this message must be specified, and here the media play a role. It is not emphasized only on a specific media, that is, a specific magazine or network is not considered, but it depends on the type of message, the audience, and the dissemination channels of access definition (P1. Line 79–82).”*

Research should have a language of conversation with people so that they can feel that scientific findings are able to solve their problems. The results must reach people from different media so that they can feel its effects in their lives. If the research output is limited only to study, it will remain in isolation, and the conditions for communication and public trust in research will not be satisfied. This will occur in the shadow of publishing findings in the language of their specific audience, integrating research processes and applying the findings in real life and different situations. One of the participants says,

*“People need to feel that research solves their problem. That is, they have to communicate. They should build public confidence in research, make research results understandable and publish messages on university websites, social networks, and public magazines (P. 6, Line 45–47)”*.

The development of communication technologies on the Internet has created a pathway of releasing outputs and communication channels through online scientific networks. These networks have now become an important gateway for sharing the scientific output, as well as discussing and exchanging knowledge and scientific topics. They have been able to attract the attention of many researchers to communicate and share the scientific knowledge because of the easy access and communication conditions provided in these networks. Many participants confirmed that this network was an approach to share and publish their research findings. The information conditions of research activities have provided researchers with working and scientific communication.

“*Publishing article is not the only good way to use results. Although we publish a dissertation or hold scientific conferences, it is not effective for the people. It should be possible to be informed through radio, television and informal channels; it should be in the public language and social media, then we should be able to use it” (P. 3, Line 46–48)”*.

### Participation of citizens in various research stages

The two concepts of citizen science and crowdsource in research are based on the participation of citizens in different stages of research, which is considered an essential component of research process in the structure of open science so that people in the society can determine the effectiveness of research output. Also, trusting and applying the results in different conditions and following the research findings leads the health system towards more research productivity so that research products are based on people’s requirements. Public participation can occur in all stages of research, from identifying problems and issues to proposing research topics to the process of research conducting and evaluating. On the other hand, people’s participation in different stages creates trust in research, making them feel self-esteem and usefulness. This participation also leads to publication, as well as timely and better application of research findings in different departments:

*“But if people take part, they can contribute to writing the report, as well as publishing and applying the results and accepting the research findings. Even people in the community feel that they are useful by participating in the research and affecting community trust in the research (P. 2, Line 47–49).”*

Sharing the results for people in the community at different levels and positions is an important component of research, which can lead to development of a research culture, the application of findings, and trust in research. In the structure of open research, it should be possible to draw the roadmap and convey the results to different audiences so that everyone can benefit from these findings to the best of their ability. The citizen science can help achieve this goal, providing the conditions for research application and sharing for members of the community through the people themselves via participating in the publication and sharing process. Another participant says,

*“The most important thing that citizen science creates, I think, is the discussion of building trust (i.e., trust and confidence). For example, in discussion on COVID-19, people did not trust the results very much because they were not transparent, but if people participate, they can also refer to other people” (P. 2, Line 46–48).*

So far, the most important form of community participation has emerged in data collection. In the context of open research, this role can be enhanced with extensive communication facilities. Using data collection platforms based on demographic work provides the conditions for the participation of many people from community in research. In most cases, this cooperation has been on the part of people in the community who play the role of research sample. Human society has been involved in most studies conducted on the health system, and the participation of members of society as the research field can be helpful in this regard.

### Concerns and reluctance to open research

To conduct research in the framework of open-science, a number of concerns were raised for researchers and managers of health system in interviews. Lack of protection of ideas in open publication and applying personal opinions is related to “prolonged review and applying personal opinions” and “loss of idea before final publication”. From the participants’ standpoint, these are concerns for which the necessary conditions and infrastructure must be established before the full implementation of open research. Since currently no system has been introduced to protect all stages of research and also provide access conditions for different people, they are reluctant to share and publish their work until the research project is completed.

Provided that systems such as clinical trial systems that exist all over the world are implemented and if it is possible to follow different stages, the conditions for implementation, monitoring, and follow-up of this type of research process will be facilitated. Thus, they will tend to use research outcomes, and doubts can be removed. Also, the current challenges from the participants’ point of view are the negative effects of open reviews and prolonged period of time needed for the publication of final findings. Numerous opinions will be applied on the outputs. In cases where the culture of research transparency and honesty is not accepted, personal opinions have a great impact on evaluations. Moreover, publishing the information of reviewers and authors will result in personal communications or consideration of personal opinions, which is a negative aspect of this process. In addition, the publication of comments and opinions of reviewers sometimes leads to misunderstandings and negative effects on research findings, which reduces the core value of the work. For example, Participant 12 stated:

*“There are many challenges in open review, and the relationships can be very effective, and in my opinion, the reviewers should not be aware of the information of authors and reviewers. I also do not agree with comments of the reviewer that are published along with the article. It seems that after real corrections, the article should be published, and the review must be without comments because it causes misunderstandings” (P. 12. Line 30–32).*

### Prolonged review and consideration of personal opinions

Review based on personal opinions is a sub-category discussing open reviews based on the opinions of participants. To launch this type of research and open review, it is necessary to deal with cultural the issue in the country first so that people understand the situation and engage in the review process without considering personal opinions and communications. Participant 14 stated:

*“The reviewers are known and we even see the names of authors; sometimes they even index the comments of reviewers, but in our country, I do not think it will be very effective. In my opinion, if the reviews are open, the situation in Iran will decline and personal opinions will be much more pronounced” (P. 14, Line 35–36).*

### Loss of idea before final printing

The lack of protective infrastructures is the next factor contributing to the reluctance for this research process in the current situation. They did not even agree with the pre-print release because the review process is long, so they lose their novelty until the final publication of the ideas; they are more inclined to release their output after the completion of the work and review process. Also, in the structure of open research, new and technological ideas may be lost, and the authors become reluctant to publish their work. In these cases, a time limit can be set, and some rights can be defined for the stakeholders to reach the desired impressions by the researchers over the specified time period. After the end of this period, they can publish the outputs. For example, Participants 9 and 12 stated:

*“It jeopardizes the interests of the individual. The interests of the individual may be endangered, especially in the technological and innovative field, and there is no mechanism to support this” (P. 9, Line 32).*

*“I myself do not agree much with the pre-print until the final work is released because it may reduce the novelty of the work, and the original idea of the work may be used by others” (P. 12, Line 10).*

## Discussion

Considering the importance of open science and its influence on publication trends of research outputs, the current study aimed to evaluate the perception of Iranian health researchers regarding the concept of open science and the impact of its methods on publication and sharing of research outputs in accordance with analysis of interviews (Table 2). The perceived concepts include access to unrestricted outputs, the use of collective wisdom in research and effective methods on publications related to free access to all types of output, increased trust in research, creation of diverse publication paths, and citizens’ participation in research stages, leading to the formation of new opportunities and challenges in health system research. This indicates that future studies should address the relationships between these factors, as well as management and optimal use of conditions created in the research structure of health system. The results revealed that new directions could be developed in the structure of open science for the research process. The impact of open science on publication and sharing process should be clarified more comprehensively and from different aspects. Studies demonstrate a variety of aspects, including the positive effects of open science techniques on increasing trust in science [16], facilitating access to research products and outputs [17], and improving the attraction of research results, as well as communication and interaction between researchers and policy makers [18]. The democratization of knowledge has been discussed through open science techniques [19]. The findings showed that health system researchers have a positive view of open science trends in the publication and sharing of research outputs, and it is therefore necessary to provide more stable conditions for using these methods in health system research via assessing different aspects.

Our first analysis of the concept of open science showed that it could pave the way for applying collective knowledge, which leads to further confidence in scientific findings and outputs. Research outputs are the result of teamwork, and in a research project in the health system, people from different fields must participate, indicating the fact that a research work is based on collective knowledge. Another function of open science is deriving maximum benefit from sharable results of research without imposing any obstacles to researchers and decision makers in health-related issues. To promote cumulative knowledge and improve research in various fields, it is therefore important to accept the open science initiative based on four principles of transparency, validity, replicability, and accessibility as a guideline for understanding research behaviors [21]. Also, population resources in medicine can lead to high quality results, widespread participation of society, and open science. Two essential elements of open science are related to availability of a large group of people, both skilled and unskilled, who propose potential solutions. The sharing of solutions is achieved through implementation of open access. People can be a central force in developmental, preclinical, and clinical research on population sources [22]. One of the important aspects of open science is that it actively involves citizens and non-experts in the research process, which can potentially benefit numerous actors, including scientists, citizens, policy makers, and the society in general [2].

Subsequent analysis of the effects of open science on publication process of research outputs has indicated that open science methods can have the greatest impact on various aspects of research publication and sharing. Approaches to the accessibility and sharing of scientific resources and knowledge are related to data management and processing, as well as analysis and communication methods for collaboration and publication of scientific resources [23]. The methods of data sharing, citing, and reusing are vital aspects in reproducibility of research [24].

Providing conditions for maximum open access to research outputs is considered a basic element of open science in research processes as well as a turning point that can provide conditions for access and optimal use of all types of research outputs. Many organizations point to the importance of archiving and long-term preservation of such archives given the power of datasets to generate new knowledge. These organizations suggested promoting the visibility of science worldwide, including for educational purposes, the general public and development of more user-friendly interfaces. Indeed, one of the main aspects of knowledge circulation is to ensure that scientific work meets the requirements of users and is discoverable, accessible, interpretable, and reusable [3].

New models of open access to scientific publications consist of many different channels and processes, including the open access publications, preprints, and alternative print platforms [25–27]. The most obvious aspect of open science is the open access journals and repositories that have facilitated the publication process and scientific communication. The application of digital tools for publishing and archiving has developed publishing methods, facilitated the access of all members of society to academic resources and expanded research-based knowledge. Acceleration of the publishing process may provide conditions for timely, appropriate access to knowledge in the digital world through journals, information centers, and various organizational repositories [26]. Further commitments on data sharing lead to greater transparency, research integrity, promotion of scientific accuracy and reproducibility to confirm research results and discover errors and plagiarism [13].

To publish outputs, access conditions must be determined based on the type of outputs and contacts, which is an important point in health-related studies. It is better to specify the protocol and a communication basis to determine the level of access and sharing according to the type of research and its outputs. Moreover, open access does not mean unlimited access, but it must be classified and systematically defined. A variety of security, economic, and political issues require the determination of access conditions systematically so that personal preferences are not applied. By implementing more transparent research methods, authors have the opportunity to start and present more replicable and valid works so that the scientific community benefits through facilitating the sharing and preservation of research materials and making new discoveries [28].

Open science requires a systematic change in current practices to create transparency, ensure the sustainability of related social and physical infrastructures and further enhance public trust in science [3]. Hence, transparency and openness should be encouraged not only by scientists and researchers but also by funding and research agencies [29]. The analysis also revealed that transparency indicates honesty and trust in research.

The research is often reliable when different stages of it are transparent. Therefore, there should be organizations or committees that provide the substrate for transparency through recording different stages of research such as protocols, initial plan, evaluation, and allocation of identifiable codes. Moreover, the researchers themselves must also report different stages of their research honestly and completely. To achieve this goal, a suitable structure or platform should be considered for research. To be published in reputable journals, clinical trial studies must first be registered in reputable data banks and registers, so that this process is considered a step to improve scientific transparency [30]. Furthermore, the publication of protocols, raw data, analysis scripts, pre-registration, clarification of financial sources, and clear expression of conflict of interest in articles are considered transparency conditions [27]. Monitoring the publication process and fulfilling the obligations of researchers lead to the publication of research products and access to them [31]. Journals have also contributed to the accuracy of studies and research outputs by sharing data sets. Publishing all positive and negative results of researchers is another important factor affecting the transparency, which will greatly reduce research errors and contribute to proper evaluation. In fact, research transparency makes researchers and stakeholders accountable for the publication and accuracy of research results. Journals should present incentives to follow open scientific methods, encourage them and clearly express their requirements [30]. Publications, pre-registration studies, the development of open science skills, formulating guidelines for promoting transparency and openness are considered the incentives for open science practices that can be useful for journals [32]. Reproducibility and transparency are important principles of the scientific process to improve efficiency, self-correction, and validity of research works [33]. Reproducibility is applicable based on reuse of open protocols, open code and data [30, 34], certification of research processes with online tools such as notebooks [35], open research methods [36], documentation of replicable calculations [37], preprint studies and reports [4, 38].

Based on the current analysis, further studies are required on how to implement the open science methods in different research processes, especially in the publication and incentive policies so that these methods are used by both researchers and stakeholders. These studies can pave the way for better application of open science methods and the accountability of stakeholders regarding the implementation, promotion, accountability, and transparency of investigations in different areas of the health system; it also facilitates the application of research findings and the relationship between these findings and readers. All these things necessitate the accompaniment of researchers with regular and detailed planning. By utilizing lessons related to the methods and principles of open research in the curricula of postgraduate studies and training of young researchers, it is possible to encourage and develop the principles of open science in the future. Evidence shows that the movement towards open science has positive results and actually makes scientists more influential in different fields [23]. Open science techniques have great potential to accelerate learning and create new knowledge, speed up the process of research and innovation to find solutions to major social challenges, fostering the growth of innovative and entrepreneurial human resources [39].

### Limitations

There are different viewpoints regarding open science as well as many doubts in using its methods in publication and sharing process of research outputs. There is a wide range of research fields in the health system; we confirm that only one part of the research community from the health system participated in the study. It is therefore recommended to evaluate and analyze different dimensions of the research fields as well as necessary considerations in open science methods from the perspective of more individuals in order to elucidate the concept of openness and its effective methods on different aspects of research to specify the necessary contemplations and policies.

## Conclusion

One of the most prominent issues expected from the structure of open science, which can be considered among its main goals, is to provide the maximum access conditions to scientific outputs for the people in the society with understandable language free from complexity. If we wish to have comprehensible outputs for the people in the society, we must simultaneously publish findings in different languages through both official and unofficial channels. In addition to scientific journals, newspapers, television, popular magazines, or other mass media can be used to publish the selection of results in the language of the public. The researcher is required to release findings in non-scientific language in one of these media, the organizations should support the research, and even the government must provide the conditions for publishing the results in the language of public. The final readers of research findings are mostly general public, especially in the field of health. As a result, we must be able to present the results in the language of its specific reader because each reader is a ring in the chain of results, and the users who should be able to benefit from the findings. Therefore, different models of scientific discourse should be implemented by researchers and related organizations, and this discourse about the research findings should reach people through different media. This is important because in the field of health, people are associated with the researcher, and the researcher’s responsibility is to present the results in the language of public. However, scientific knowledge cycle and circulation are needed to properly benefit from the research output and create conditions for scientific discourse, the results of which will present more incentives for researchers, helping different industries and improving the level of health and well-being in the society. Moreover, the correct cycle of research based on sharing outputs and the participation of people in different stages of research leads to more trust in research structures and further participation, resulting in the effectiveness of research on different social sectors.

## Data Availability

The datasets formed and analysed during the current study are available from the corresponding author on reasonable request.
